# Novel Drug Candidate Prediction for Intrahepatic Cholangiocarcinoma via Hub Gene Network Analysis and Connectivity Mapping

**DOI:** 10.3390/cancers14133284

**Published:** 2022-07-05

**Authors:** Yao Xiao, Baoluhe Zhang, Jordan M. Cloyd, Laura Alaimo, Gang Xu, Shunda Du, Yilei Mao, Timothy M. Pawlik

**Affiliations:** 1 Department of Liver Surgery, Peking Union Medical College Hospital, Chinese Academy of Medical Sciences, Beijing 100730, China; xiaoy12@mails.tsinghua.edu.cn (Y.X.); zblh6001@gmail.com (B.Z.); dushd@pumch.cn (S.D.); maoyilei@pumch.cn (Y.M.); 2 Department of Surgery, The Ohio State University Wexner Medical Center and James Cancer Hospital and Solove Research Institute, Columbus, OH 43210, USA; jordan.cloyd@osumc.edu (J.M.C.); laura.alaimo@osumc.edu (L.A.); 3 Department of Liver Surgery and Liver Transplant Center, West China Hospital of Sichuan University, Chengdu 610040, China; gangxu@wchscu.cn

**Keywords:** intrahepatic cholangiocarcinoma, differentially expressed genes, weighted gene co-expression network analysis, drug prediction, connectivity map, biliary tract cancer

## Abstract

**Simple Summary:**

Only about 15% of intrahepatic cholangiocarcinoma (ICC) patients meet the criteria for resection at the time of diagnosis. For patients with advanced and/or metastatic disease, the development of novel therapeutic strategies is urgently needed. The aim of our study was to identify possible novel therapeutic targets and drugs for ICC by using transcriptomic profiles from the Gene Expression Omnibus databases and The Cancer Genome Atlas. The weighted co-expression gene network was constructed to screen hub genes. Potential drug candidates with promise in the treatment of ICC were identified by analyzing key protein–protein interaction (PPI) networks of the hub genes to identify potential interacting drugs based on the Connectivity Map database.

**Abstract:**

Intrahepatic cholangiocarcinoma (ICC) is an aggressive malignancy, and there is a need for effective systemic therapies. Gene expression profile-based analyses may allow for efficient screening of potential drug candidates to serve as novel therapeutics for patients with ICC. The RNA expression profile of ICC and normal biliary epithelial cells were downloaded from the Gene Expression Omnibus (GEO) and The Cancer Genome Atlas (TCGA) databases. Function annotation and enrichment pathway analyses of the differentially expressed genes (DEGs) were finished using the Gene Ontology (GO) and Kyoto Encyclopedia of Genes and Genomes (KEGG) databases. A weighted gene co-expression network (WGCN) was constructed by WGCN analysis (WGCNA). Key genes from the DEGs and co-expression gene modules were analyzed to generate a protein–protein interaction (PPI) network. The association between the top 10 screened hub genes and the overall and disease-free survival of ICC patients was examined. The Connectivity Map (cMap) analysis was performed to identify possible drugs for ICC using hub genes. A total of 151 key genes were selected from the overlapping genes of 1287 GSE-DEGs, 8183 TCGA-DEGs and 1226 genes in the mixed modules. A total of 10 hub genes of interest (CTNNB1, SPP1, COL1A2, COL3A1, SMAD3, SRC, VCAN, PKLR, GART, MRPS5) were found analyzing protein–protein interaction. Using the cMap, candidate drugs screened with potential efficacy for ICC included three tyrosine kinase inhibitors (dasatinib, NVP-BHG712, tivantinib), two cannabinoid receptor agonists (palmitoylethanolamide, arachidonamide), two antibiotics (moxifloxacin, amoxicillin), one estrogen receptor agonist (levonorgestrel), one serine/threonine protein kinase inhibitor (MK-2206) and other small molecules. Key genes from network and PPI analysis allowed us to identify potential drugs for ICC. The identification of novel gene expression profiles and related drug screening may accelerate the identification of potential novel drug therapies for ICC.

## 1. Introduction

Intrahepatic cholangiocarcinoma (ICC) is an aggressive biliary tract cancer (BTC) originating from the biliary epithelium distal to the secondary biliary radicals. ICC is the second most common liver malignancy, accounting for about 10% of all cholangiocarcinomas [1]. Over the past decade, there have been extensive efforts towards advancing the diagnosis, staging, classification and treatment of ICC [2]. Complete surgical resection remains the only curative treatment for ICC with an estimated median survival ranging from 27 to 36 months [3,4,5]. However, as an insidious malignancy with latent symptoms, only about 15% of ICC patients meet the criteria for resection at the time of diagnosis [1]. In addition, for patients with advanced and/or metastatic disease, the development of novel therapeutic strategies is urgently needed [4].

Cancer genomics such as transcriptomic analyses have accelerated the implementation of precision medicine by identifying new therapeutic targets [6]. Establishing cancer-specific differentially expressed gene profiles to screen potential drug candidates rapidly and efficiently has been proposed to identify novel therapies. To this end, the Connectivity Map is a database containing over 1.5 M gene expression profiles from ~5000 small-molecule compounds and ~3000 genetic reagents that has been tested in multiple cell types [7]. Weighted correlation network analysis (WCGNA) can be used to identify co-expressed gene modules and explore gene networks and the phenotypes of interest, as well as core genes in a multi-profile network. WCGNA also facilitates network-based gene screening to predict candidate diagnostic and prognostic biomarkers, as well as potential therapeutic strategies [8,9]. Using a multi-database approach to validate key gene networks and identify potential therapeutic targets has been employed in diseases such as head and neck squamous cell carcinoma (HNSCC), hepatocellular carcinoma (HCC), esophageal carcinoma and extrahepatic cholangiocarcinoma [10,11,12,13,14]. In addition, drug target prioritization using mapping custom gene expression profiles in conjunction with drug-gene profile cMap networks has become increasingly utilized [15].

Intrahepatic cholangiocarcinoma has a particularly poor prognosis, even among patients who undergo curative-intent resection [16,17]. To date, traditional systemic agents such as gemcitabine, cisplatin, and oxaliplatin have only modest activity [18,19,20]. While recent data have suggested some benefits with adjuvant capecitabine, novel systemic therapies are urgently needed to improve the outcomes of patients with advanced ICC [21]. The objective of the present study was to recognize possible innovative targets and drugs to treat ICC by means of transcriptomic profiles from the Gene Expression Omnibus (GEO) databases and The Cancer Genome Atlas (TCGA). The weighted co-expression gene network was constructed to screen hub genes. Potential drug candidates with promise in the treatment of ICC were identified by analyzing key protein–protein interaction (PPI) networks of the hub genes to identify potential interacting drugs based on the cMap database.

## 2. Materials and Methods

### 2.1. Database Selection and Preprocessing

**Figure 1:**
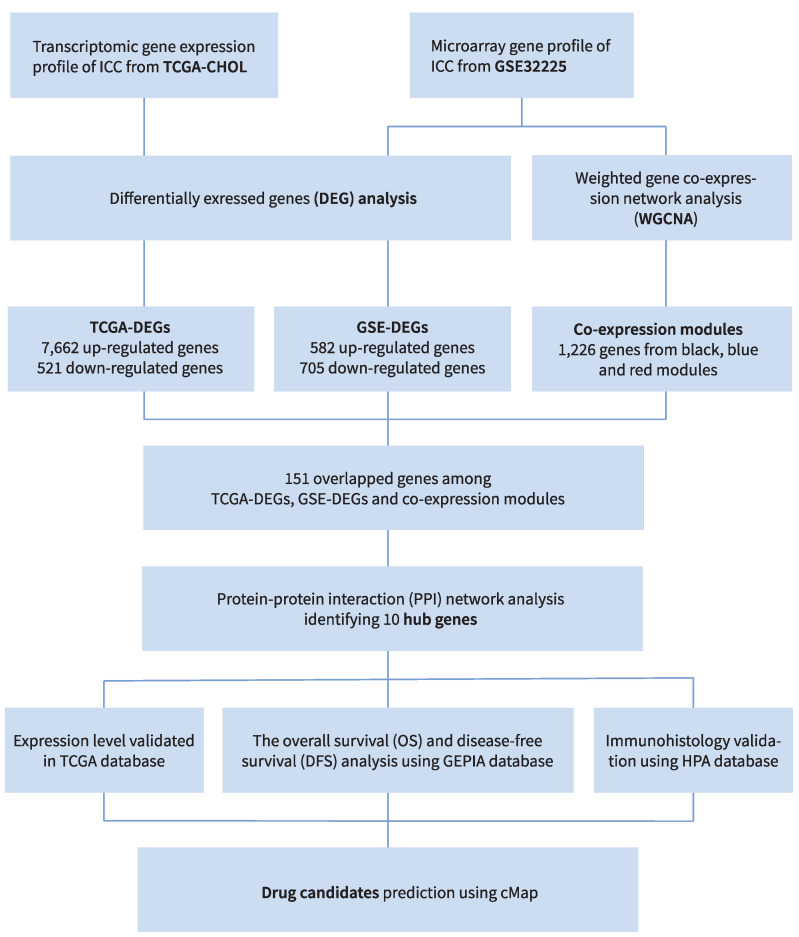
Study design and workflow.

The workflow is shown in Figure 1. The expression gene profile array associated with ICC was obtained from GSE32225 on the GEO (Available online: http://www.ncbi.nlm.nih.gov/geo (accessed on 20 March 2022)) [22]. The gene expression profile was acquired by high-density single nucleotide polymorphism array using 149 formalin-fixed samples from ICC patients and 6 normal biliary epithelial cell controls [22]. The raw data included data from all platforms, samples and GSE records, and were acquired as MINiML formatted family files. The data were processed by Log2 transformation and normalized by quantiles function (preprocessCore package in R software). The platform annotation information of the normalized data guided the probes conversion into gene symbols. The mean of probes corresponding to two or more genes was calculated. The principal component analysis (PCA) chart allowed the visualization of the batch effect, which was removed by removeBatchEffect function of limma package of R software. Box plots were created to evaluate the level of standardization of the data according to the results of preprocessing [23,24,25].

The transcriptomic gene expression profiles and clinical data of ICC in the TCGA database were downloaded by R package TCGAbiolinks from the TCGA-CHOL project [26]. A total of 44 samples with a primary site of liver and intrahepatic bile ducts were downloaded and classified into 35 ICC tumor samples and 9 non-tumor normal tissues. The edgeR and EDAseq packages were used to complete data outlier filtering (correlation cutoff = 0.6), normalization, and data filtering (quantile cutoff = 0.25).

### 2.2. Differentially Expressed Gene, Function Annotation and Enrichment Analysis

The R package limma and edgeR were applied to the GSE32225 and TCGA-CHOL, respectively, to analyze the differentially expressed genes (DEGs) between ICC and normal samples in each dataset. In GSE32225 DEG analysis, the Benjamini–Hochberg method was used to adjust the *p*-value for a control of the false discovery rate (FDR). Genes with a cutoff of |logFC| ≥ 1.0 and adj. *p* < 0.05 were defined as DEGs. In TCGA-CHOL dataset, glmLRT method was used, with cutoff |logFC| ≥ 1.0 and FDR < 0.05 to generate the DEGs. The DEGs of both datasets were visualized as a volcano plot by using the ggplot2 package [27].

The reference for function annotation of the DEGs was Human gene GRCh38 [28]. The functional and pathway enrichment knowledge of the DEGs were provided by Gene Ontology (GO, available online: http://geneontology.org/ (accessed on 20 March 2022)) and Kyoto Encyclopedia of Genes and Genomes (KEGG, available online: http://www.kegg.jp/ (accessed on 20 March 2022)) databases. Three items are classified by GO: molecular function (MF), biological process (BP), cell composition (CC). KEGG database includes a systematic analysis of genome information and the corresponding roles of genes. ClusterProfiler was applied for GO annotation and KEGG enrichment analysis [29].

### 2.3. Co-Expression Modules by WGCNA

WGCNA identifies genes with very high correlation or sets of genes that share similar functions, called modules, and allows correlation analysis of modules with phenotypic data to uncover potential hub genes. The gene expression data profiles of GSE32225 were selected for gene co-expression network construction using the WGCNA package [8]. A soft-thresholding power *β* = 4 was built by the function powerEstimate. An intermediate quantity called the co-expression similarity *s*_*ij*_ was first defined, and then the adjacency matrix *a*_*ij*_ was decided by the formula *a*_*ij*_ = *s*_*ij*_^*β*^. A topological overlap matrix (TOM) and hierarchical clustering dendrogram of the 1-TOM matrix was generated and gene modules were identified by unsupervised clustering. Sample tree and log-log plot of whole-network connectivity was generated to visualize the gene distribution in each module. The module–trait relationships between modules and clinical trait (tumor vs. normal sample) information were also determined by the method described by Wang et al. [30]. The modules with top 3 high correlation coefficients were considered most relevant to clinical traits and were grouped together for downstream analysis.

### 2.4. Key Gene Selection, Protein–Protein Interaction Analysis, and Hub Gene Screening

The top 3 clinical trait-correlated modules were named the mixed modules. The overlapping genes between GSE-DEGs, TCGA-DEGs and co-expression genes extracted from mixed modules were used to screen key genes by a Venn diagram. The key genes were put into Basic Local Alignment Search Tool (Blastx, available online: https://blast.ncbi.nlm.nih.gov/ (accessed on 20 March 2022)) analysis to find the corresponding homologous proteins. The interaction network among these proteins was then analyzed by the Search Tool for the Retrieval of Interacting Genes/Proteins (STRING) database (Available online: https://string-db.org/ (accessed on 20 March 2022)) [31]. Importing the PPI data into Cytoscape (version 3.8.2) software, genes with high interaction in candidate modules, which is the most key gene subnetwork in the PPI map, were identified as hub genes [32]. The degree-based topology algorithm was used by CytoHubba to analyze the network and choose the top 10 hub genes and their sub-networks to additionally create a key node network [33].

### 2.5. Hub Gene Survival Analysis and Identification of Potential Drug Candidates

The differentially expressed profile of each hub gene in ICC was compared using boxplot in Gene Expression Profiling Interactive Analysis (GEPIA) [30]. The association with outcomes was examined by assessing overall (OS) and disease-free (DFS) survival using the log-rank test. The different protein expression levels were compared between ICC and cholangiocytes in normal liver using The Human Protein Atlas (HPA) (proteinatlas.org). The median gene expression of all samples was set as the cohort threshold. The cox proportional hazard ratio and the 95% confidence interval was also calculated. Potential curative drugs for ICC were collected from the Connectivity Map (cMap, available online: https://clue.io/ (accessed on 21 March 2022)) [7]. In order to explore the potential efficacy of these drug candidates for ICC, the corresponding pharmacologic effects were depicted by searching for the published clinical relevance on National Institutes of Health database (Available online: https://pubchem.ncbi.nlm.nih.gov/compound (accessed on 7 April 2022)).

## 3. Results

### 3.1. Identification of Differentially Expressed Genes in GSE32225 and TCGA-CHOL

**Figure 2:**
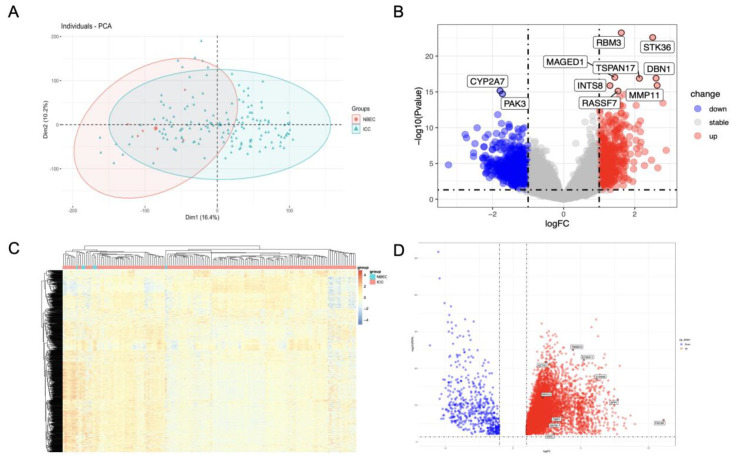
DEG analysis. (**A**) PCA plot of GSE32225 dataset. Green dot represents the ICC sample, red dot represents the normal biliary epithelial cells. (**B**) Volcano plot of GSE-DEGs constructed using log2FC values and adjusted *p*-value. Red and blue points in the plot represent the up-regulated and down-regulated genes defined as described. (**C**) Hierarchical cluster heatmap of GSE-DEGs. (**D**) Volcano plot of TCGA-DEGs.

A threshold *p*-value of <0.05 and |log2FC| > 1 was set to recognize DEGs from GSE32225 (GSE-DEGs). Duplicates were removed and the investigation of raw data of 18,402 genes was performed detecting genes upregulated and downregulated in the tumor tissue (*n* = 582 and *n* = 705, respectively). PCA plot and a volcano map of the DEGs were created to visualize the different patterns of profile expression between normal and tumor tissues (Figure 2A,B). The details of DEGs clustering were identified by the heatmap (Figure 2C).

DEGs from the TCGA-CHOL project (TCGA-DEGs) were identified based on a threshold |logFC| ≥ 1.0 and FDR < 0.05. Raw data of 60,660 genes were processed, revealing 7662 up-regulated and 521 down-regulated genes. A volcano map of the TCGA-DEGs was plotted to visualize the DEG distribution (Figure 2D).

### 3.2. Function Annotation and Enrichment Analysis of GSE-DEGs

**Figure 3:**
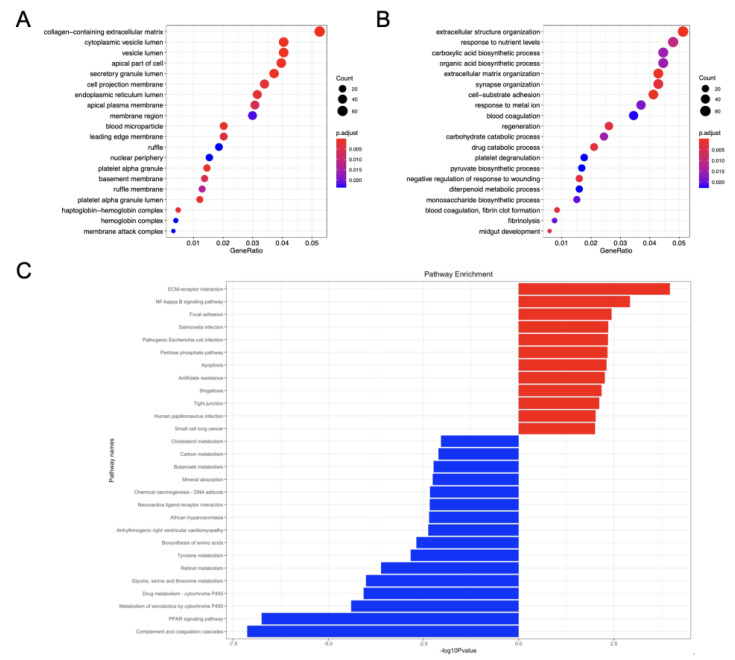
Analysis of function and enrichment of GSE-DEGs. (**A**) CC category of GO annotation diagram of GSE-DEGs. (**B**) BP category of GO annotation diagram of GSE-DEGs. X-axis: enrichment factor (ratio of number of entries in the foreground gene set to number of background genes enriched in the same entry). Y-axis: enrichment pathways. The number of genes in the pathway determines dot size in direct proportion. The color of the dots (from red to blue) corresponds to the q-value reduction in sequence, although the difference is more significant. (**C**) Bar chart representing KEGG enrichment: enriched pathways of up-regulated genes (red) and enriched pathways of down-regulated genes (blue).

A significant gene enrichment of DEGs between ICC and normal biliary epithelial cells was demonstrated by KEGG and GO analyses of functional enrichment for 1287 GSE-DEGs. Therefore, bubble charts allowed to choose and visualize the top 20 pathway entries with significant enrichment. Figure 3A,B display the most enriched GO annotations related to cell component (CC) and biological process (BP). The results of CC reveal that the GSE-DEGs were mostly linked to the collagen-containing extracellular matrix, cytoplasmic vesicle lumen, vesical lumen, the apical part of the cell and secretory granule lumen. The most enriched BP entries were associated with extracellular structure organization, response to nutrient levels, the carboxylic acid biosynthetic process, the organic acid biosynthetic process as well as extracellular matrix organization. No MF entries were significantly enriched.

A total of 28 enriched KEGG pathways (12 associated with upregulated genes and 16 with downregulated genes) were identified by KEGG enrichment analysis and pathway screening using a *p*-value < 0.01 (Figure 3C). The upregulated pathways were linked to ECM–receptor interaction, NF-κB signaling pathway, focal adhesion, Salmonella infection and Escherichia coli infection. The downregulated pathways included complement and coagulation cascades, PPAR signaling pathway, metabolism of xenobiotics by cytochrome P450, drug metabolism and glycine, serine and threonine metabolism.

### 3.3. Construction of Weighted Gene Co-Expression Modules

**Figure 4:**
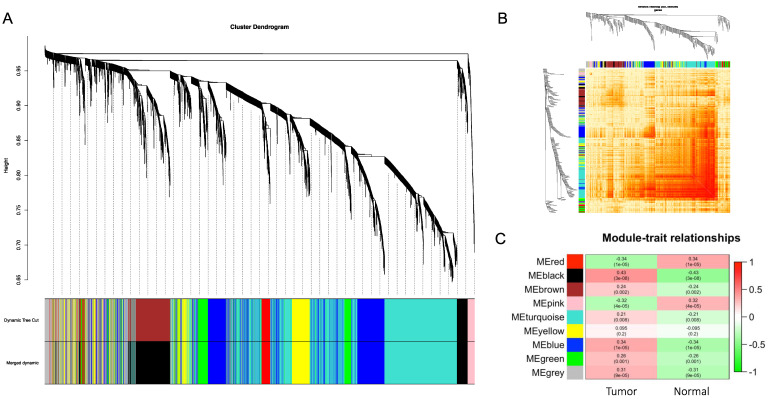
Weighted gene co-expression network. (**A**) Gene dendrogram obtained by average linkage hierarchical clustering. The color row underneath the dendrogram shows the module assignment determined by the Dynamic Tree Cut. (**B**) Network heatmap plot. Branches correspond to modules in the hierarchical clustering dendrograms. Darker color indicates higher co-expression and interconnection. (**C**) Module–trait relationships. X-axis: clinical trait of tumor or normal. Y-axis: clustered modules. The correlation and *p*-values are labeled in each block.

The weighted gene co-expression network was constructed from the GSE32225 dataset with the WGCNA package to identify the functional clusters in ICC. A total of nine modules were identified with each module assigned to a color (Figure 4A). The co-expression network was visualized in the network heatmap plot (Figure 4B). Except for the gray module, which represents genes that were not assigned into any cluster, the other eight modules were plotted to the heatmap of module–trait relationships to evaluate the association between each module and the clinical traits (tumor and normal) (Figure 4C). The highest correlation with tumor was identified in the black and blue modules, while the red module had the highest correlation with normal tissues (black module: r = 0.43, *p* = 3 × 10^−8^; blue module: r = 0.34, *p* = 1 × 10^−5^; red module: r = 0.34, *p* = 1 × 10^−5^).

### 3.4. Survival Gene Screening, PPI Analysis, and Hub Gene Selection

**Figure 5:**
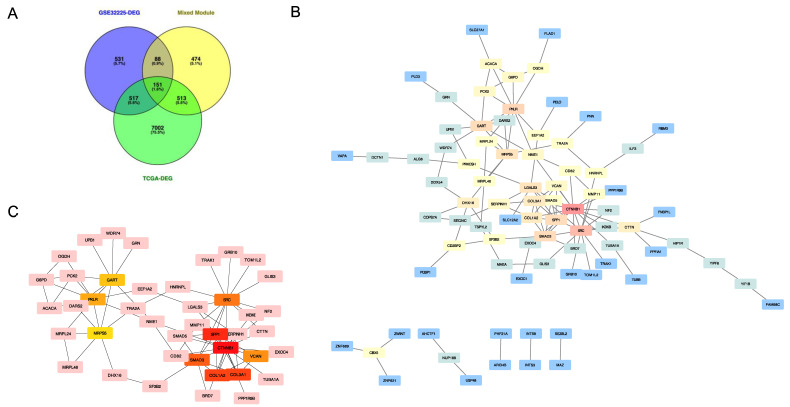
Identification of top 20 hub genes. (**A**) Venn diagram of GSE-DEGs, TCGA-DEGs and mixed module. (**B**) Protein–protein interaction network generated by the overlapped genes in three datasets. Red: high score; orange-yellow: intermediate score; blue: low score. (**C**) Protein–protein interaction network among 10 hub genes generated by *Cytohubba*. Greater scores in degree algorithm and relevant location in the network are represented by darker colors.

In order to screen genes of interest, the overlapping genes among 1287 GSE-DEGs, 8183 TCGA-DEGs and mixed modules containing 1226 co-expression genes extracted from the black, blue, and red modules were identified and visualized in the Venn diagram (Figure 5A). A total of 151 key genes were selected. The means of the STRING with confidence >0.4 and FDR stringency of 0.05 were used to assess the PPI network associated with these key genes. A PPI network including 150 nodes, 118 edges and an average node degree of 1.57 was produced. The STRING network was examined applying the Cytoscape for hub genes screening (Figure 5B). The top 10 hub genes were calculated from the PPI network using the degree algorithm plugin cytoHubba: CTNNB1, SPP1, COL1A2, COL3A1, SMAD3, SRC, VCAN, PKLR, GART, MRPS5 (Table 1). Figure 5C shows the PPI connections among the hub genes.

**Figure 6:**
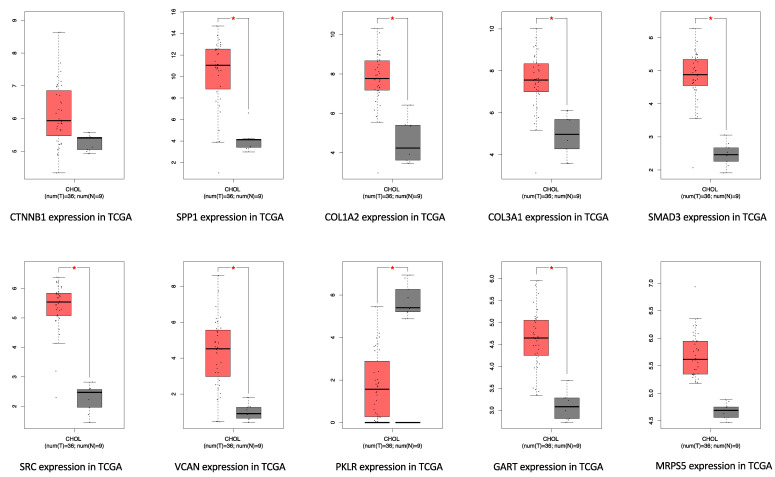
Gene expression level of top hub genes in TCGA-CHOL. Red box: tumor sample; gray box: normal tissue. log2(TPM + 1) scale was used to transform the expression data for plotting.

**Figure 7:**
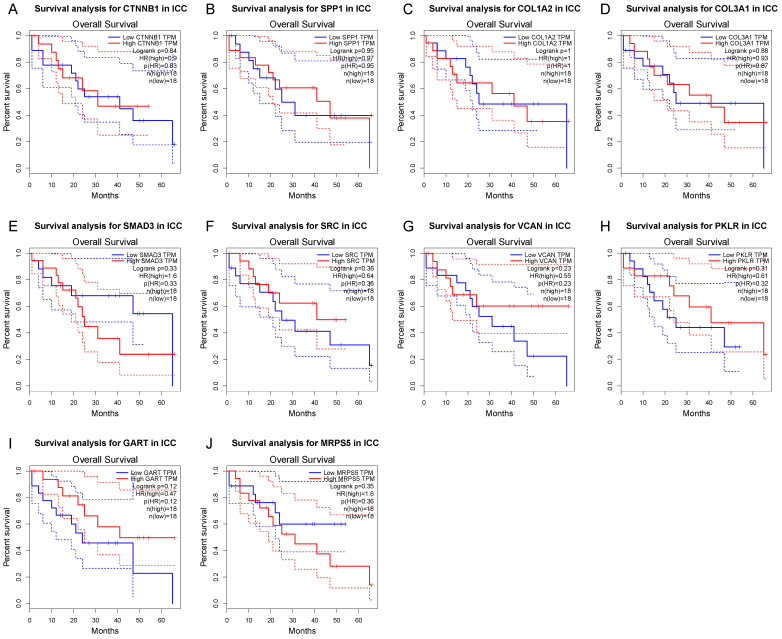
Kaplan–Meier curves of hub gene expression and overall survival for each of the top hub genes. Blue curve: low-expression group; red curve: high-expression group.

**Figure 8:**
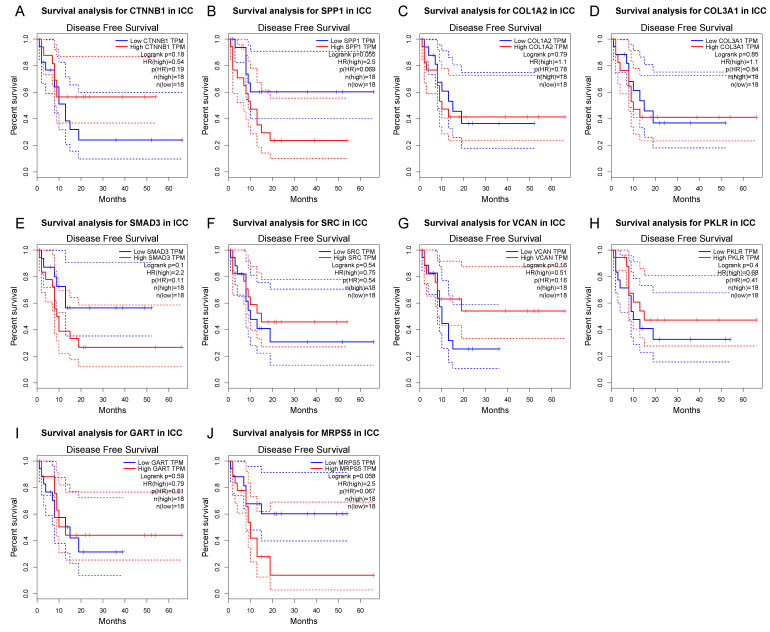
Kaplan–Meier curves of hub gene expression and disease-free survival for each of the top hub genes. Blue curve: low-expression group; red curve: high-expression group.

**Figure 9:**
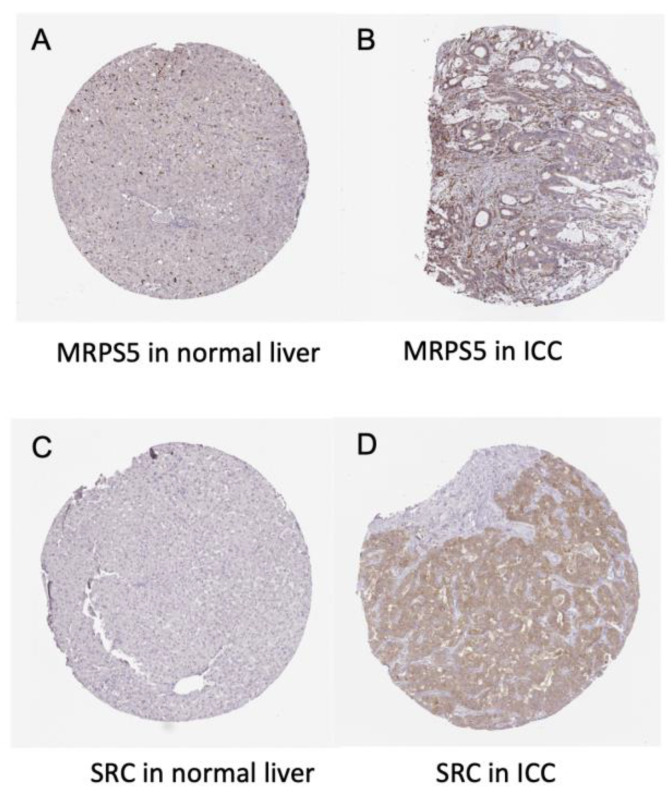
Immunohistochemistry of the MRPS5 and SRC genes in ICC and normal liver tissues. (**A**) MRPS5 in normal liver (cholangiocyte staining: not detected; intensity: negative; quantity: none). (**B**) MRPS5 in ICC (staining: low; intensity: weak; quantity: 75–25%; location: cytoplasmic and membranous). (**C**) SRC in normal liver (cholangiocyte staining: not detected; intensity: negative; quantity: none). (**D**) SRC in ICC (staining: medium; intensity: moderate; quantity > 75%; location: cytoplasmic and membranous).

The differential expression of the 10 hub genes in the TCGA cohort were then compared using GEPIA (Figure 6). Among these 10 hub genes, all but PKLR were up-regulated in ICC, which was consistent with the different expression in the GSE32225 database. OS and DFS related to these top hub genes from the TCGA clinical database were also analyzed by GEPIA, respectively, to examine the prognostic value of these genes (Figure 7 and Figure 8). No gene demonstrated significant impact on OS or DFS relative to the expression levels. In addition to exploration at the transcriptional level, the protein levels of the hub genes were also searched in The Human Protein Atlas database to validate the relationship between the protein product and ICC. SRC and MRPS5 had significantly higher expression in ICC samples compared with normal cholangiocytes in liver (Figure 9).

### 3.5. Potential Drug Candidates of ICC-Related Gene Profile

The cMap database contains a wide range of small-molecule libraries, and their experimental effect is tested on various common cell lines, enabling the users to generate a hypothetic compound list of research interest. According to the manual, the weighted connectivity map score is an algorithm used to compute the potential gene expression profile when each drug is treated on a specific cell line. In the query function, the user can import a list of up- or down- regulated genes differentially expressed in each group (cancer vs. normal, gene knockout vs. wildtype, etc.) under desired experiment design. A drug-cell line combination that shares a highly similar gene expression profile with the input query gives a high positive connectivity score, while a high negative score indicates a dissimilarity or opposite effect. Thus, when it comes to DEGs in cancer, a high positive score of a drug indicates its potential to induce the cell to have a cancer-like gene expression, while a high negative score implicates a potential opposite therapeutic effect. In the cMap database, the 10 hub genes were input as an index of regulated genetic profile of ICC. Candidate drugs with the top 27 highest negative scores by a cutoff raw score of 0.7 were screened (Table 2).

## 4. Discussion

ICC is a highly lethal malignancy that has an increasing incidence [34,35,36]. Advances in surgical management, locoregional therapy, as well as the development of some targeted therapies have improved the treatment of patients with ICC. For example, for the subset of patients with localized disease, surgical resection may be a curative-intent treatment option [37,38]. Unfortunately, most patients present with advanced disease at the time of diagnosis [39], and patients who undergo resection frequently experience early recurrence [16]. As such, improving outcomes for patients with ICC will require advances in systemic therapy. To date, several targeted therapies have been approved for the treatment of advanced ICC. In particular, targeted therapy has been approved for the roughly 15–20% of patients with FGFR2 fusions/rearrangements/deletions, as well as a smaller subset of patients with IDH1 mutations or dMMR [39,40,41]. Unfortunately, only a small minority of patients with ICC have these specific targetable genetic perturbations and are eligible for these therapies. As such, there is an ongoing need to identify pathogenic mechanisms involved in ICC tumorigenesis to inform future novel systemic treatment options.

The rapid advance of sequencing technology has allowed a deeper analysis and understanding of the molecular mechanism of ICC, thus promoting progress in the research on molecular diagnosis and targeted therapy. To that end, in the current study, we used a bioinformatics gene mapping technique to identify deleterious genes specific to ICC. In addition, we then screened drug candidates to identify potential pathways that warrant further investigation of therapeutic effect in ICC. Specifically, differentially expressed genes between ICC samples and normal biliary epithelial cells in GSE32225 and TCGA-CHOL datasets were identified; these data were then used to construct a weighted co-expression gene network by WGCNA. The function annotation and enrichment analysis of the GSE-DEG were examined. Overlapping genes among the three blocks were picked as key genes to establish a PPI network. A total of 10 hub genes were calculated from the network and their association with OS and DFS of ICC patients was examined. In addition, the gene–clinical trait connections were also validated in protein level by the IHC staining of ICC and normal cholangiocytes in HPA. Hub genes were then selected based on interacting perturbagens to generate a list of drug candidates. Of note, three of the top scored drug candidates were tyrosine kinase inhibitors (dasatinib, NVP-BHG712, tivantinib) and two were cannabinoid receptor agonists (palmitoylethanolamide, arachidonamide). Other drug candidates included two antibiotics (moxifloxacin, amoxicillin), one estrogen receptor agonist (levonorgestrel), and one serine/threonine protein kinase inhibitor (MK-2206). The other two were related to cellular biochemical regulation processes, involving one lipid peroxidase inhibitor (anisodamine) and one dihydrofolate reductase inhibitor (pyrimethamine).

The tyrosine kinase family is a large multigene subtype with particular relevance to multiple human diseases, especially cancer. Dasatinib is a multikinase inhibitor, which has been demonstrated to have a favorable therapeutic effect in many malignant diseases [42]. In fact, dasatinib has been reported to have hypersensitivity in ICC with isocitrate dehydrogenase (IDH1/IDH2) mutations [43]. This study identified SRC as a critical dasatinib target in IDH mutant ICC. Collectively, data from the current study, as well as early clinical information from previous reports, suggest that additional dasatinib may be a promising agent in the treatment of ICC [43]. In separated studies, tivantinib, an antiproliferative agent, has been proposed as second-line therapy for hepatocellular carcinoma following disease progression after sorafenib treatment based on receptor overexpression analyses [44]. In fact, data from phase I and II trials have noted promising results [45,46]. In a phase II randomized placebo-controlled trial (NCT00988741), patients treated with tivantinib not only had an increased median OS (7.2 vs. 3.8 months) and RFS (2.2 vs. 1.4 months) versus placebo, but also had an increased median time to progression (TTP) (2.7 vs. 1.4 months) [46]. However, in a phase III study, tivantinib did not lead to improved overall survival [47]. Given the findings of the current study, as well as preliminary HCC data, the clinical application of tivantinib for ICC warrants additional studies to determine its therapeutic effect relative to different targeted biomarkers.

Cannabinoids (CB) have demonstrated the effect to reduce tumor-associated symptoms such as neuropathic pain [48]. Some reports have explored the role of CBs in tumor proliferation and progression, suggesting that CBs interact with CB-Rs, thereby inducing an anti-cancer effect [49]. Endocannabinoids work via particular G-protein-coupled receptors (GPRs), CB-Rs (CB1-R and CB2-R), in which CB2-R has an established role in carcinogenesis and cancer progression [50]. In fact, CBs have been proposed as novel anti-tumor targets in patients with non-small cell lung cancer (NSCLC) and breast cancer [50,51]. While the mechanism remains poorly defined, palmitoylethanolamide (PEA) is an endogenous fatty acid amide related to CB-Rs that facilitates an anti-inflammatory effect [52]. In fact, ultra-micronized PEA (um-PEA) has been shown to inhibit tumor cell proliferation and tumor cell migration both in vitro and in vivo [52]. As such, further investigation of CB-R agonists and their therapeutic anti-proliferative effect relative to ICC may be a potential area to explore.

Estrogen receptors (ERs) are widely expressed in many cell types involved in both innate and adaptive immune responses. ERs are composed of two subtypes: ERα and ERβ. Both receptors interact similarly with endogenous estrogens and play a role in the development and progression of multiple malignant diseases, including breast, prostate, endometrial, ovarian, colorectal and lung cancers [53,54]. Levonorgestrel is usually administered by an intrauterine device that releases consistent localized progestin for the treatment of endometrial cancer [55]. Many studies have shown that ERs are generally expressed in cholangiocytes and demonstrate up-regulation during cell proliferation [56]. The fact that female sex has been previously reported as risk factor for ICC is consistent with the role of estrogen in ICC pathogenesis [57,58]. As such, the targeting of ER pathways may mitigate ICC oncogenesis and future studies will be necessary to examine further the inner connection between ERs and ICC progression.

Our study yielded a new hypothesis that some drug families including tyrosine kinase inhibitor, cannabinoid receptor inhibitor and estrogen receptor inhibitor, may be investigated as candidate drugs for ICC treatment. Due to the heterogeneity and complexity of ICC as well as many other tumors, lack of response to targeted therapies, and frequent existence of drug resistance, the need for novel drug candidate has been largely increased [59]. Ideally, both computational methods and experimental methods should be applied to screen potential drugs. In our study, WCGNA and drug-drug interactions derived computational methods were utilized to help study molecular signature differences between tumor and normal tissue, as well as different classes of drugs in the context of ICC targeted therapy. The approaches we applied in our study can serve as more rapid and effective means for screening and identifying candidate drug combinations for ICC. To achieve better data quality, a series of bioinformatic and statistical methods have been applied in sequencing data interpretation and connectivity map to overcome data noise in biological networks and literature mining results [7,59].

Interpretation of data from the current study should be interpreted in light of several limitations. While data from the current study are important to focus screening and identification of candidate drug combinations to treat ICC, development of any new drug therapies requires further experimental validation and preclinical tests before it can be applied to a clinical scenario. While both computational methods and experimental methods ultimately need to be applied to screen potential drugs, the current study provides the framework for future experiments. In particular, computational screening allows for the identification of potential therapies and better direction/application of future experiments in an informative and cost-saving way. For example, WCGNA and drug-drug interactions derived from computational methods can direct future studies aimed at molecular signature differences between different classes of drugs in specific disease contexts.

## 5. Conclusions

In conclusion, using an integrated approach with DEGs of ICC from the GEO and TCGA databases, together with clinical trait-associated gene modules, a key hub gene network was generated to evaluate protein-level expression in ICC versus normal tissue samples. Analyzing the hub genes, the drug candidate list of small molecules was created, and these could possibly be used as classes of novel pharmacologic agents for future studies to identify and develop innovative drugs for ICC treatment.

## Figures and Tables

**Table 1:** Top 10 hub genes produced by protein–protein interaction network analysis.

Rank	Name	Score
1	CTNNB1	83
2	SPP1	66
3	COL1A2	50
3	COL3A1	50
5	SMAD3	39
6	SRC	25
7	VCAN	24
8	PKLR	17
9	GART	11
10	MRPS5	10

**Table 2:** Candidate drugs and their mechanism of action identified via connectivity mapping.

Drug Name	Tested Cell Line	Test Dose	Test Time	Mechanism of Action	Raw Connectivity Score
Levonorgestrel	A549	10 uM	24 h	Estrogen receptor agonist|Glucocorticoid receptor antagonist|Progesterone receptor agonist|Progesterone receptor antagonist	−0.79
Nicotinamide	HEPG2	10 uM	6 h	Protein synthesis stimulant	−0.75
Palmitoylethanolamide	A549	0.04 uM	24 h	Cannabinoid receptor agonist	−0.74
Arachidonamide	HCC515	10 uM	6 h	Cannabinoid receptor agonist	−0.74
Moxifloxacin	PC3	1.11 uM	24 h	Bacterial DNA inhibitor	−0.74
Anisodamine	HELA	10 uM	24 h	Lipid peroxidase inhibitor	−0.74
Dasatinib	SHSY5Y	0.04 uM	24 h	KIT inhibitor|Bcr-Abl inhibitor|Ephrin inhibitor|PDGFR inhibitor|Src inhibitor|Tyrosine kinase inhibitor	−0.73
NVP-BHG712	CD34	10 uM	24 h	Ephrin inhibitor	−0.73
Pyrimethamine	HT29	10 uM	24 h	Dihydrofolate reductase inhibitor	−0.73
CO-101244	VCAP	10 uM	6 h	Ionotropic glutamate receptor antagonist	−0.72
MK-2206	HME1	20 uM	24 h	AKT inhibitor	−0.71
Phenamil	HA1E	10 uM	24 h	TRPV antagonist	−0.71
Tivantinib	A549	10 uM	24 h	Tyrosine kinase inhibitor	−0.71
Amoxicillin	HCC515	10 uM	6 h	Penicillin binding protein inhibitor	−0.71
MDL-28170	HCC515	10 uM	6 h	Calpain inhibitor	−0.71
Pyrimethamine	MCF7	10 uM	24 h	Dihydrofolate reductase inhibitor	−0.71
Salvinorin-A	HT29	0.25 uM	24 h	Opioid receptor agonist	−0.71
Varenicline	NPC	0.74 uM	24 h	Acetylcholine receptor agonist	−0.71
FR-180204	A549	10 uM	24 h	MAP kinase inhibitor	−0.71
Flunisolide	A549	10 uM	24 h	Cytochrome P450 inhibitor	−0.71
PNU-22394	HT29	10 uM	6 h	Serotonin receptor agonist	−0.7
Tianeptine	A375	10 uM	6 h	Selective serotonin reuptake inhibitor (SSRI)	−0.7
Dazoxiben	MDAMB231	2.22 uM	24 h	Thromboxane synthase inhibitor	−0.7
CDC	MCF7	10 uM	6 h	Lipoxygenase inhibitor	−0.7
BRL-37344	NPC	10 uM	24 h	Adrenergic receptor agonist	−0.7
Ursolic-acid	A375	70 uM	24 h	11-beta-HSD1 inhibitor|Acetylcholinesterase inhibitor|ATPase inhibitor|Caspase inhibitor|HIV protease inhibitor|Lipid peroxidase inhibitor|NFKB inhibitor|Quorum sensing signaling modulator|STAT inhibitor|Steryl sulfatase inhibitor|Tyrosine phosphatase inhibitor	−0.7
Meglitinide	YAPC	0.08 uM	24 h	Potassium channel antagonist	−0.7

## Data Availability

All data analyzed in this paper can be found in: TCGA (https://portal.gdc.cancer.gov/), GEO (https://www.ncbi.nlm.nih.gov/geo/), GEPIA (http://gepia.cancer-pku.cn/), HPA (https://www.proteinatlas.org/), cMap (https://clue.io/).
